# Deteriorating fractional exhaled nitric oxide preceding pneumonitis during immune checkpoint inhibitor therapy: a case report

**DOI:** 10.1016/j.rmcr.2026.102501

**Published:** 2026-09-28

**Authors:** M. Högman, J. Ellingsen, Y. Naeser, A.T. Dinh-Xuan

**Affiliations:** a Department of Medical Sciences, Respiratory, Allergy and Sleep Research, Uppsala University, Uppsala, Sweden; b Department of Oncology, University Hospital, Uppsala, Sweden; c Department of Immunology, Genetics and Pathology, Uppsala University, Sweden; d Department of Respiratory and Sleep Medicine, Cochin Hospital, University Paris Cite, Paris, France

**Keywords:** Melanoma, Fractional exhaled nitric oxide, Pulmonary toxicity, Immune checkpoint inhibitor, Immune-related adverse event, COPD

## Abstract

Immune checkpoint inhibitors (ICIs) such as nivolumab have transformed the management of metastatic cutaneous malignant melanoma but are associated with immune-related adverse events, including pulmonary toxicity. Standard monitoring of ICI therapy commonly includes computed tomography (CT) scans, blood tests, and clinical assessment. We report a 78-year-old man with metastatic malignant melanoma treated with nivolumab whose fractional exhaled nitric oxide (Fe_NO_) was monitored longitudinally. Baseline Fe_NO_ had been stable at 40-50 ppb for a decade. After initiation of nivolumab, Fe_NO_ rose progressively, exceeding 100 ppb after seven cycles and surpassing 250 ppb after 13 cycles, preceding radiological and routine laboratory abnormalities. Fe_NO_ transiently decreased with inhaled corticosteroid therapy. A CT scan after 19 cycles showed pulmonary opacities and small amount of bilateral pleural effusions consistent with ICI-related pneumonitis. Oral corticosteroids promptly normalised Fe_NO_ and blood inflammatory markers, and the radiological abnormalities resolved. A milder recurrence occurred seven months later and again responded to systemic corticosteroids. In this patient, Fe_NO_ elevation preceded conventional indicators of pulmonary toxicity during ICI therapy. These observations support further evaluation of Fe_NO_ as a potential early biomarker for ICI-related pulmonary toxicity.

## Highlights

• Fe_NO_ elevation preceded conventional indicators of pulmonary toxicity during ICI therapy.
• Fe_NO_ monitoring before, during and after ICI therapy may provide an early indicator of immune-related pneumonitis.
• Fe_NO_ measurement is non-invasive, rapid, and reproducible, and patients can serve as their own control.
• Asthma and COPD are associated with an increased risk of IRP, and screening for elevated Fe_NO_ levels before immunotherapy could be beneficial.

## Abbreviations

COPD: chronic obstructive pulmonary disease
CRP: C-reactive protein
CT: computed tomography
Fe_NO_: fractional exhaled nitric oxide
ICIs: immune checkpoint inhibitors
ICS: inhaled corticosteroids
iNOS: inducible nitric oxide synthase
irAEs: immune-related adverse events
IRP: immune-related pneumonitis
mCMM: metastatic cutaneous malignant melanoma
NCS: nasal corticosteroids
OCS: oral corticosteroids

## 1 Introduction

Over the past decade, immune checkpoint inhibitors (ICIs) have revolutionised the treatment of metastatic cutaneous malignant melanoma (mCMM) by enhancing the immune system's ability to recognise and eliminate cancer cells. Despite their impressive clinical efficacy, ICIs frequently cause immune-related adverse events (irAEs) resulting from excessive immune activation. A potentially fatal irAE is immune-related pneumonitis (IRP), which was reported in 14% of mCMM patients in one study, with chronic IRP in 2% of cases [1]. In another study of patients with mCMM, IRP occurred in 5% of patients, and recurrent pneumonitis despite permanent discontinuation of ICI therapy occurred in 1% [2].

Monitoring during ICI therapy typically includes blood tests, assessment of clinical symptoms, and imaging as indicated. Disease status and treatment outcomes are usually evaluated by imaging.

The production of nitric oxide is driven by upregulation of inducible nitric oxide synthase (iNOS) and is induced by proinflammatory cytokines. Fractional exhaled nitric oxide (Fe_NO_) is an inflammatory biomarker commonly used in asthma management when T2 inflammation is present and strongly associated with eosinophil infiltration and activation. Eosinophilic inflammation has been reported in several case reports during ICI therapy: one describing chronic rhinosinusitis in the treatment of renal cell carcinoma [3], another reporting new-onset asthma in the treatment of endometrial cancer [4], and a third case report with lung adenocarcinoma with the development of chronic obstructive pulmonary disease (COPD) [5]. In those reports, ICIs were administered for 3, 5 and 13 months, respectively, and Fe_NO_ values ranged from 84 to 136 ppb, baseline Fe_NO_ was not provided. A level of Fe_NO_ > 50 ppb supports a diagnosis of asthma. In stable COPD, about one third had Fe_NO_ values above 19 ppb (median 32 ppb, IQR 27-50), with no major changes over time [6]. A recent review summarises Fe_NO_ metabolism in COPD [7].

Immune-related pneumonitis ranges from mild to life-threatening and is treated with systemic corticosteroids using a step-down tapering regimen. Patients with concomitant COPD appear to have a higher risk of IRP [8]. A meta-analysis concluded that among lung cancer patients, those with COPD had an increased risk of developing IRP during ICI therapy (odds ratio 1.54; 95% CI 1.24-1.92; p < 0.01) [8]. A retrospective observational study suggests that IRP gets more severe in patients with asthma than in those without [9].

## 2 Case report

A 78-year-old male with no history of asthma, allergies, or tobacco use presented to his primary care physician with respiratory symptoms, notably difficulties with forced expiration. Evaluation for a possible COPD diagnosis was initiated. Only one spirometry was performed in primary care. It was abnormal, showing an obstructive pattern with a forced expiratory flow at 1 s/forced vital capacity (FEV_1_/FVC) ratio of 0.63. Consequently, a computed tomography (CT) scan was performed. During this assessment, a pulmonary metastasis from mCMM was identified (Fig. 1a). The patient subsequently began treatment with the ICI nivolumab, 480 mg intravenously every 4 weeks.

**Fig. 1:**
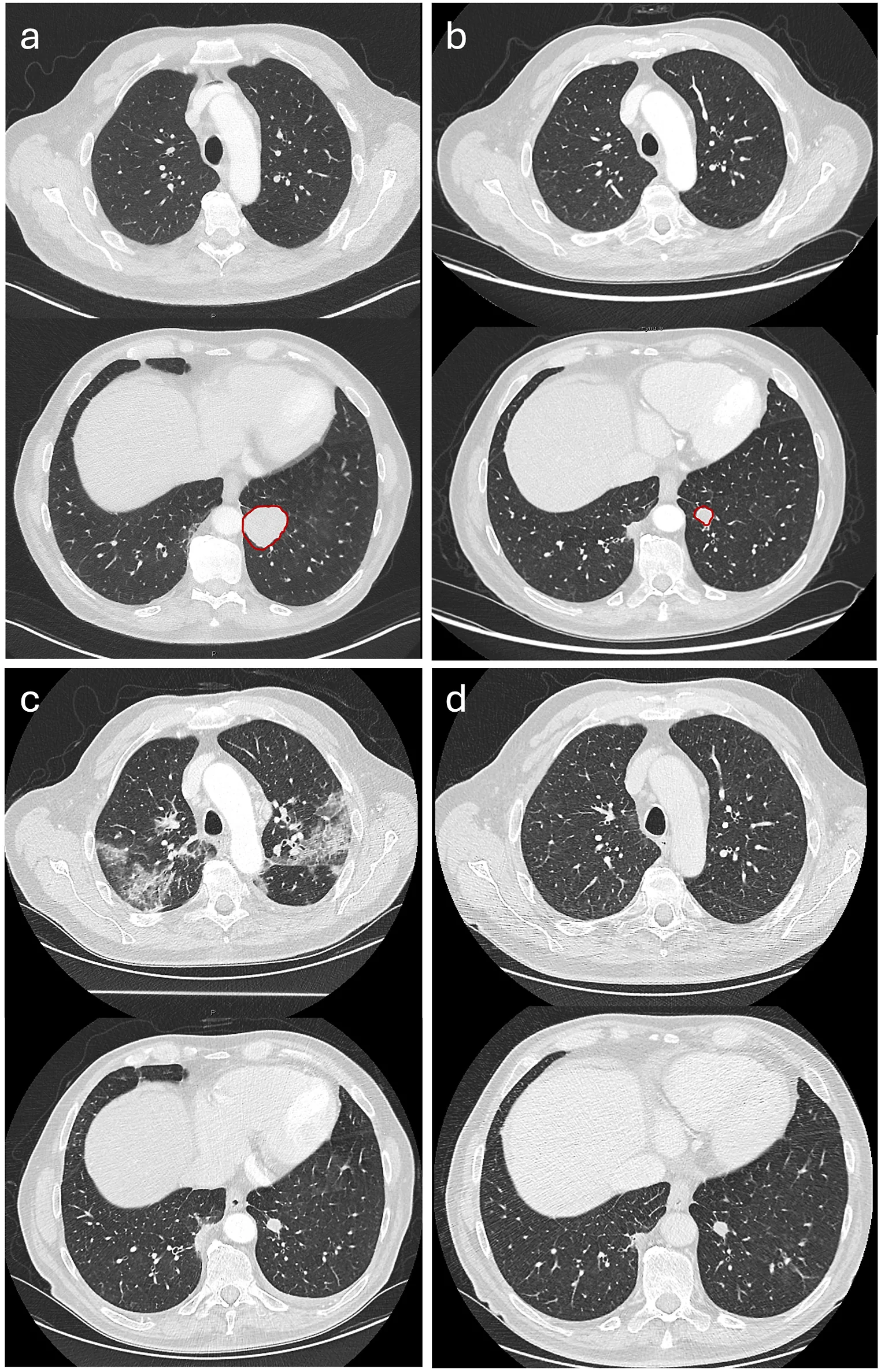
Chest computed tomography scans: a) before nivolumab therapy, with the largest pulmonary metastasis marked, b) after 9 cycles of nivolumab, demonstrating reduction of metastasis size, c) immune-related pneumonitis after 19 cycles of nivolumab, d) resolution of pneumonitis after oral prednisolone.

The patient himself, a research engineer with a long-standing interest in NO, measured Fe_NO_ in addition to standard clinical monitoring and shared the measurements with his oncology team. Fe_NO_ was measured at a flow rate of 50 mL/s, in accordance with the ATS/ERS 2005 recommendations, using an electrochemical sensor (NIOX Vero, NIOX Group plc, Oxford, UK). Over the preceding 10 years, baseline Fe_NO_ had consistently ranged between 40 and 50 ppb. After initiation of nivolumab, Fe_NO_ rose steadily (Fig. 2). After seven cycles, the patient reported nasal congestion, and Fe_NO_ exceeded 100 ppb. A routine CT scan on treatment day 254 showed widespread opacities in the frontal, ethmoid, and sphenoid sinuses, with no abnormalities in the lung parenchyma. An otolaryngologist diagnosed rhinosinusitis and started intranasal corticosteroids, but Fe_NO_ continued to rise, exceeding 250 ppb after 13 cycles. Blood test at that time showed a normal neutrophil count (6.8 × 10^9^/L) and mild eosinophilia (1.0 × 10^9^/L). During this period, the primary skin CMM tumour was surgically excised, and the largest pulmonary metastasis decreased in maximum diameter from 5.7 cm to 1.7 cm (Fig. 1b). Routine oncology monitoring revealed no other significant adverse effects, and nivolumab treatment continued.

**Fig. 2:**
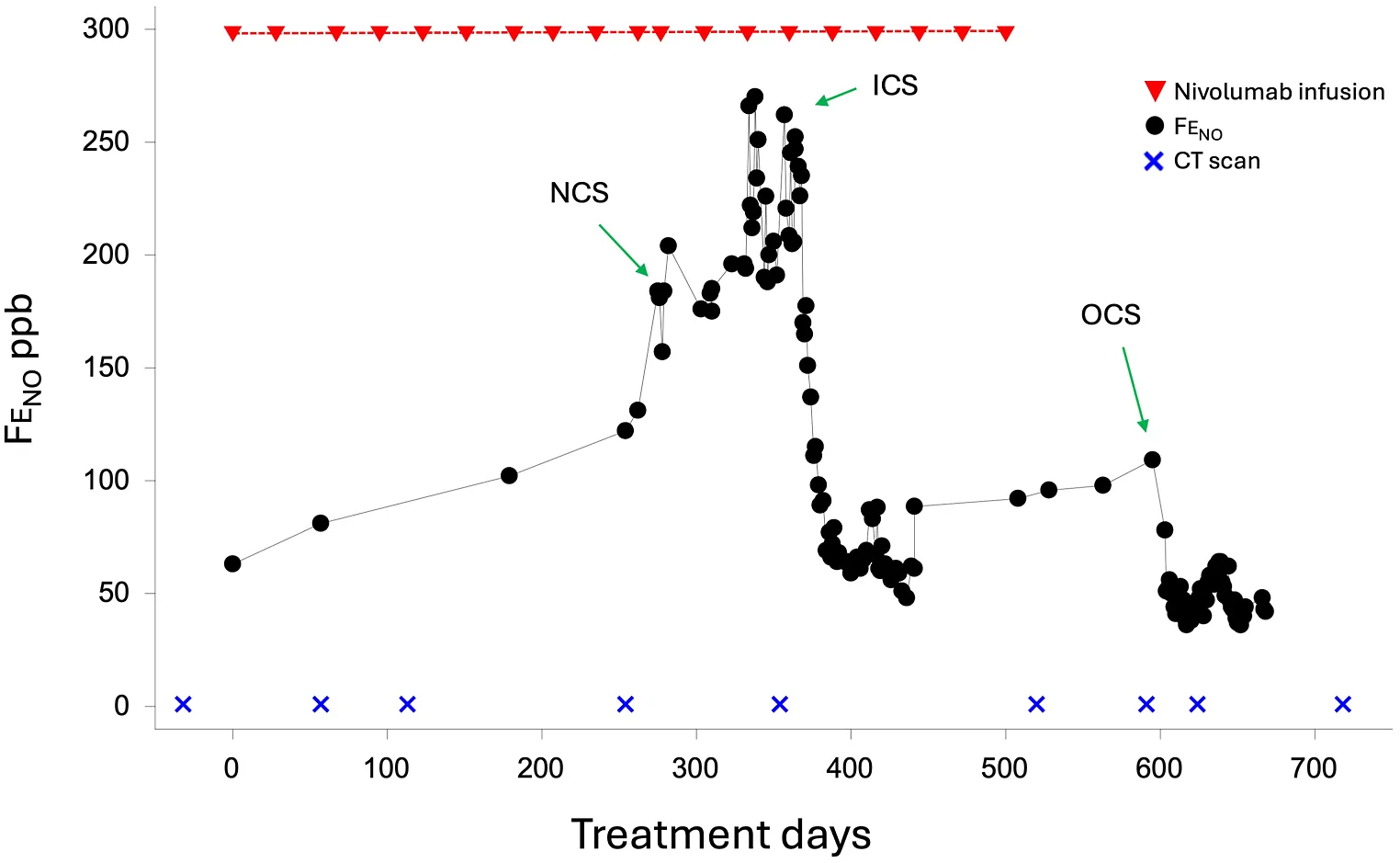
Fe_NO_ measurements over nivolumab treatment cycles, with the development of nasal congestion after 11 cycles. Nasal corticoid steroids (NCS) did not stop Fe_NO_ levels from rising. After 14 cycles, inhalation therapy with a combination of inhaled corticosteroids (ICS) and a long-acting beta-2 agonist was started, and a prompt decrease in Fe_NO_ to baseline levels was observed. After 17 cycles of nivolumab, Fe_NO_ levels began to rise gradually. Due to progression of respiratory symptoms, nivolumab infusion was stopped after 500 treatment days. A CT scan on treatment day 591 (Fig. 1c) showed irAE, and oral corticosteroids (OCS) were started. Fe_NO_ returned to baseline levels, and the irAE resolved on treatment day 624.

Because Fe_NO_ remained persistently elevated, with progression of respiratory symptoms (productive cough, dyspnoea, nasal congestion), and a CT scan on treatment day 354 showed no change in lung metastases and no new suspicious findings, the patient was referred to a pulmonologist. The patient was started on inhaled corticosteroids (ICS) combined with a long-acting beta-2 agonist (LABA), after which Fe_NO_ returned to baseline (Fig. 2). Despite ongoing inhaled therapy, Fe_NO_ rose again after 17 cycles of nivolumab to >100 ppb and respiratory symptoms worsened. A CT scan after 19 cycles (treatment day 520) demonstrated stable disease. On treatment day 591, the CT scan revealed new-onset patchy pulmonary opacities and small bilateral pleural effusions.

Fe_NO_ values began to rise on treatment day 442. Plasma C-reactive protein (CRP), total leukocyte count, and neutrophil count showed a steady increase beginning on treatment day 525 (Table 1). The radiology and the clinical presentation were consistent with IRP (Fig. 1c), and nivolumab was permanently discontinued. Oral corticosteroids (OCS) were started (prednisolone 50 mg) with a step-down tapering regimen, which promptly normalised Fe_NO_ to baseline (Table 1, Fig. 2). CRP returned to normal levels by the end of the OCS course. Follow-up CT scan showed complete resolution of the pulmonary and pleural abnormalities (Fig. 1d).

**Table 1:** Change in Fe_NO_ with the corresponding blood analysis at the first immune-related pneumonitis (IPR). Computed tomography scan detected IPR on treatment day 565 (T565) with nivolumab (Figs. 1c), 93 days after the first Fe_NO_ rise. Treatment with OCS was started T601, 129 days after the first rise in Fe_NO_. The second IRP occurred 336 days after the last nivolumab infusion.

Treatment days	Fe_NO_ ppb	CRP mg/L	Leukocytes 10^9^/L	Neutrophils 10^9^/L
First irAE				
T442	61	0.4	9.1	5.8
T472	88	0.5	8.4	5.6
T525	97	2.4	12.4	8.4
T570	98	27	13.9	6.7
T596	109	57	15.5	7.1
T628 (OCS of 27 days)	44	77	8.5	7
T656 (OCS of 58 days)	36	0.6	11.4	8.3

Second irAE				
T847	59	0.8	9.6	6.8
T862	78	44	13.3	8.9
T878 (OCS of 16 days)	45			
T891 (OSC of 29 days)	40			

Abbreviations: OCS, oral corticosteroids; Fe_NO_, exhaled nitric oxide; CRP, C-Reactive Protein.

Seven months later, at 336 days after the last nivolumab infusion, a routine CT scan showed pulmonary opacities in the upper lobes consistent with IRP grade 1-2. The patient reported only a slight increase in respiratory symptoms. At the second IRP, Fe_NO_ showed a small increase on treatment day 847 and a larger increase on day 862, which coincided with elevated CRP, leukocyte and neutrophil levels (Table 1). OCS were re-initiated (prednisolone 40 mg) with a gradual taper over 6 weeks. During OCS therapy, Fe_NO_ returned to baseline and stabilised around 40-50 ppb.

Two months later, ICS/LABA inhalations were stepped down since there was no clear diagnosis mandating continued treatment. During the tapering, Fe_NO_ began to rise. Consequently, the ICS/LABA dose was increased, with a subsequent return of Fe_NO_ to baseline.

## 3 Discussion

This case report describes that increasing Fe_NO_ preceded clinically detected pneumonitis during ICI therapy for mCMM in a never-smoking male with no history of asthma or chronic rhinosinusitis. Fe_NO_ began to rise after 7 treatment cycles and reached very high levels after 13 cycles. These elevated Fe_NO_ values were accompanied by a mild peripheral eosinophilia.

Fe_NO_ is strongly associated with eosinophilic airway inflammation, which is common in allergic and chronic inflammatory conditions. Patients with asthma may be at increased risk of more severe IRP [9], and patients with COPD may be at increased risk of any pulmonary irAE [10]. However, COPD has also been associated with greater benefit from ICIs in some retrospective studies [11]. A multicentre prospective study in patients with NSCLC reported a statistically significant increase in Fe_NO_ after 4 cycles of nivolumab in COPD patients. However, the median difference in Fe_NO_ was below 10 ppb, which, in most instances, is of no clinical significance [12].

In our patient, Fe_NO_ was the earliest biomarker to signal an irAE event, preceding clinical and radiological findings by several weeks. The Fe_NO_ rise could reflect T2 cytokine activity (interleukin 5, 4, and 13), which promotes eosinophil recruitment and survival, but there may be additional sources of iNOS upregulation. Expression of iNOS in airway epithelial cells can also be induced by inflammatory cytokines outside the T2 pathways, e.g., interferon-γ, tumour necrosis factor α, and interleukin 17 [13,14]. Furthermore, interleukin 17 promotes NO-dependent expression of programmed death-ligand 1 [15], thereby linking non-T2 inflammation to NO overproduction and checkpoint-mediated immune control of tumorigenesis. Although the exact mechanism underlying the increased Fe_NO_ in the present case is unknown, we hypothesise that, in addition to T2 inflammation as evidenced by the eosinophilia, it may also be linked to iNOS upregulation mediated by non-T2 inflammatory pathways. When Fe_NO_ was at its peak, a mild eosinophilia was present, and ICS decreased Fe_NO_ to the patient's baseline levels. Despite ICS, the Fe_NO_ started to rise before the first and second irAE events. Fe_NO_ primarily reflects epithelial NO production in the airways, so an early inflammatory response may underlie the observed changes. How this airway signal relates mechanistically to IRP requires further investigation.

Fe_NO_ measurement is non-invasive, rapid, and reproducible with portable electrochemical devices, and patients can, through serial measurements, serve as their own controls. In this case, Fe_NO_ rose well before conventional clinical monitoring detected abnormalities, consistent with other case reports linking high Fe_NO_ to subsequent IRP [16]. These features make Fe_NO_ an attractive candidate biomarker for early detection of airway inflammation during ICI therapy. Our patient had elevated Fe_NO_ values before the nivolumab therapy and could therefore have been more sensitive to developing IRP. The risk of IRP seems to be increased in patients with airway inflammation, such as asthma and COPD. Future studies should evaluate whether screening for elevated baseline Fe_NO_ prior to immunotherapies is beneficial.

## 4 Conclusion

In summary, this case suggests that serial Fe_NO_ monitoring before, during and after ICI therapy may provide an early indicator of IRP. The elevation in Fe_NO_ preceded conventional monitoring abnormalities and clinically significant adverse effects, ultimately prompting treatment discontinuation and OCS therapy. These observations support further prospective evaluation of Fe_NO_ as a potential biomarker for ICI-related pulmonary toxicity.

## CRediT authorship contribution statement

**M. Högman:** Writing – review & editing, Writing – original draft, Supervision, Project administration, Methodology, Formal analysis, Data curation, Conceptualization. **J. Ellingsen:** Writing – review & editing, Visualization, Formal analysis, Data curation. **Y. Naeser:** Writing – review & editing, Validation, Formal analysis. **A.T. Dinh-Xuan:** Writing – review & editing, Methodology, Formal analysis.

## Ethics

The patient had signed an informed consent for a national project to follow the treatment effect of ICIs. However, the fraction of exhaled nitric oxide was not included in the application to the Swedish Ethical Review Authority; therefore, the patient provided informed consent to share his information.

## Consent for publication

The patient had read this case report and provided consent for its publication.

## Funding

None.

## Declaration of competing interest

The authors declare that they have no known competing financial interests or personal relationships that could have appeared to influence the work reported in this paper.
